# Influence of Build Angle and Polishing Roughness on Corrosion Resistance of 316L Stainless Steel Fabricated by SLM Method

**DOI:** 10.3390/ma15114020

**Published:** 2022-06-06

**Authors:** Hao Wang, Xiaoyong Shu, Jianping Zhao

**Affiliations:** 1 School of Mechanical and Power Engineering, Nanjing Tech University, Nanjing 211816, China; wh_njtech@163.com (H.W.); czaqzy@163.com (X.S.); 2 Jiangsu Key Lab of Design and Manufacture of Extreme Pressure Equipment, Nanjing 211816, China

**Keywords:** 316L, SLM, surface roughness, electrochemical experiments, corrosion resistance

## Abstract

Metal parts formed by laser additive manufacturing methods usually have large surface roughness, which affects the corrosion resistance of the parts. This study reported the reason for and mechanism of the large surface roughness of 316L stainless steel samples manufactured by selective laser melting (SLM) at different build angles. Through the study, the reason for the large top surface roughness (average surface roughness is 15.3 μm) is due to the molten channel structure formed on the surface. The large side surface roughness (average surface roughness is 19.1 μm) is due to the incomplete fused particles adhering to the surface. Through electrochemical experiments, the influence of the build angle and polishing treatment on the corrosion resistance of the sample was studied. The different roughness of the top and side surfaces results in different corrosion resistances (the top surface pitting potential is 0.317 $V_{Ag/AgCl}$ and the side surface pitting potential is 0.148 $V_{Ag/AgCl}$), and polishing can improve the surface corrosion resistance of specimens by reducing the surface roughness, especially for the side surface (from 0.148 to 0.351 $V_{Ag/AgCl}$). Therefore, parts manufactured by SLM can be post-treated to reduce roughness and improve surface corrosion resistance.

## 1. Introduction

Selective laser melting (SLM) is a technology that adopts metal powder to be formed by complete melting, cooling and solidification under the thermal action of a laser beam. Based on the principle of discrete stacking, and based on the digital model file, this technology realizes near-net forming by accumulating the material layer by layer. It has the advantages of simple formation, strong flexibility, high machining accuracy and a short processing cycle, and has a great developmental prospect. At the same time, SLM technology has many disadvantages, such as a high processing cost, easy internal porosity, unstable performance, etc. However, it has unique technological advantages in certain fields, such as turbine blades in aerospace, aero-engine parts, custom cooling molds, nuclear power plant equipment, lightweight design of auto parts and personalized customization in the medical field. With the rapid development of SLM technology in recent years, it has been widely used [1,2,3,4].

316L stainless steel has good toughness, corrosion resistance and low cost [5]. It is very stable in water, air and a variety of hydrochloric acid solutions, and is widely used in aerospace, nuclear power, biomedicine, shipbuilding, automotive, electronic communications, instrumentation and other fields. At present, answering the question of how to improve the mechanical properties of 316L stainless steel material has become the goal of many scholars. Many studies have shown that specimens formed by SLM usually have higher yield strength and hardness than forgings and castings [6,7]. Itziar Tolosa et al. [6] studied the mechanical properties of 316L stainless steel manufactured by selective laser melting (SLM) technology. Through experimental study, it was found that compared with the forged samples, the yield strength of SLM samples was significantly improved, while maintaining high ductility and notch impact resistance. K.Wei et al. [7] found that the hardness of the parts formed by laser additive manufacturing technology was higher than that of the casting parts of the same material, which was mainly caused by grain refinement, solid solution strengthening and content increase in the hard phase of the material. However, due to the characteristics of the SLM forming process itself, it is easy to produce microcracks, pores and other microdefects inside the forming parts. H. Meier et al. [8] conducted relevant research on SLM forming 316L stainless steel, showing that too little laser power, or too large laser scanning spacing, laser scanning speed and layer thickness will lead to more holes forming inside the forming parts, and these micro-defects will have adverse effects on the performance of the parts. Sun et al. [9] studied the wear resistance and corrosion resistance of 316L stainless steel parts manufactured by SLM. The results show that porosity is the main factor affecting the wear resistance and intergranular corrosion resistance. Metal parts manufactured by SLM not only have microdefects, such as voids and microcracks, but also usually have large surface roughness. Alrbaey et al. [10] found that the *Ra* value of a 316L part formed by SLM was 12.4 ± 3 μm. The surface roughness will affect the fatigue performance and corrosion resistance of the parts. Chola Elangeswaran et al. [11] studied the influence of machining on the fatigue behavior of a 316L part manufactured by SLM. It is found that the roughness of the formed parts can be greatly reduced by machining, and the fatigue performance can be improved by reducing the roughness. The process parameters in the SLM forming process have a great influence on the microdefects and surface roughness of the parts. The performance of the formed parts can be improved by optimizing the process parameters. Yang et al. [12] systematically changed the laser power, scanning speed, layer thickness and inclination angle, and used a surface roughness tester to measure the arithmetic average roughness *Ra* value of the surface, and studied the mechanism of roughness formation of the upper and lower inclined surfaces. Andreas Gebhardt et al. [13] studied the influence of the direction of parts forming in a cabin and the main process parameters on the surface quality of the formed parts. Giovanni Strano et al. [14] studied the surface roughness and morphology of 316L stainless steel parts formed by SLM. A surface profilometer and a scanning electron microscope were used to analyze the surfaces of the parts with different tilt angles formed by laser selective melting, and the ladder effect and top surface viscous powder effect were considered. A new mathematical model is proposed to predict the surface roughness of parts at different tilt angles. Liverani et al. [15] studied the effects of laser power, scanning speed, scanning spacing and processing direction on the microstructure and mechanical properties of 316L stainless steel samples formed by SLM, and established the optimal process parameter combination for these parameters. Li et al. [16] studied the effect of process parameters on porosity, and the results showed that the porosity increased significantly with the increase in scanning speed. Gu and Shen [17] increased the sample density by increasing the laser power, reducing the scanning speed or reducing the thickness of the powder layer. E Liverani et al. [15] found that the process parameters of SLM had a great influence on the mechanical properties and microstructure of the 316L sample. Kruth et al. [18] studied the influence of process parameters on the microstructure and mechanical properties of 316L stainless steel parts. Tolosa et al. [6] studied the influence of different forming angles on the mechanical properties of 316L stainless steel parts formed by selective laser melting, and obtained parts with mechanical properties close to those of forgings. These studies show that changing the process parameters in the SLM manufacturing process can directly affect the microstructure, surface roughness and mechanical properties of the parts. In this study, the influence of build angle on the surface roughness of the 316L samples manufactured by SLM, as well as the influence of surface roughness and polishing treatment on the corrosion resistance of the samples, were studied.

## 2. Experimental Procedures

### 2.1. Materials and Additive Manufacturing

The powder used in the SLM process was a commercially available 316L powder that was gas-atomized in an argon gas environment. The particle size distribution of the powder is listed in Table 1. The nominal composition of the 316L powder is listed in Table 2. The SLM process is printed in a chamber filled with argon to avoid oxidation of the molten pool. The processing parameters are listed in Table 3. A reciprocating printing strategy was applied. In the SLM process, a “67° turning between layers” scanning strategy was applied.

**Figure 1:**
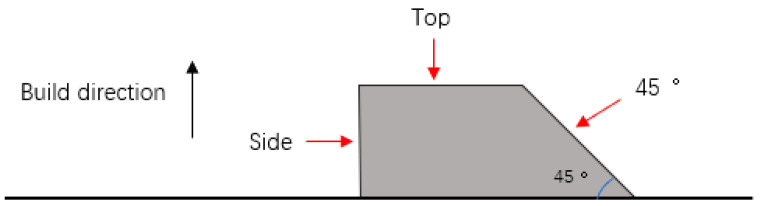
The printing method of the sample and the schematic diagram of the top surface, 45° surface and side surface.

In order to investigate the effect of build angle on surface roughness and corrosion resistance, the sample was printed as shown in Figure 1. The surface whose normal line is parallel to the build direction is labeled as the top surface. The surface whose normal line is 45° from the build direction is labeled as the 45° surface. The surface whose normal line is perpendicular to the build direction is labeled as the side surface.

### 2.2. Surface Roughness Measurement

A Olympus, DSX-CB optical microscope (Olympus Corporation, Tokyo, Japan) was used to measure the surface roughness of as-printed and polished samples. Roughness values corresponding to average surface roughness (*S_a_*) and maximum surface peak/depth (*S_z_*) were extracted. Eight different positions on each sample were measured and their averages were taken to ensure reproducibility of the measurements. Here, *S_a_* represents the arithmetic mean deviation of regional topography. *S_z_* defines the sum of the maximum peak height and maximum valley depth in a region.

### 2.3. Polishing Processing

In order to study the effect of polishing treatment on the corrosion resistance of the samples, the samples were wet-ground with 2000 SiC paper successively, degreased with alcohol, cleaned with water, and then dried in cold air.

### 2.4. Electrochemical Study

The electrochemical behavior of the as-printed and polished samples was measured using an electrochemical workstation (CHI660E). The specimen size for the electrochemical test was $15\times 15\times 5{\;\mathrm{mm}}^{3}$. A flat cell with three electrodes was set up using the specimen (with 1.0 ${\mathrm{cm}}^{2}$ corroded areas) as a working electrode (WE), a platinum wire as a counter electrode (CE), and Ag/AgCl as a reference electrode (RE). The electrochemical measurements were conducted in 6 wt.% NaCl solution (7.5 g solid NaCl was dissolved in 250 mL of distilled water) at room temperature [19,20]. The samples were immersed in the solution for one hour and the open circuit potential (OCP) was detected. The polarization test was conducted with a scan rate of 0.002 V/s, and a potential range between −1 V/Ag/AgCl and 1.5 V/Ag/AgCl. The electrochemical impedance spectroscopy (EIS) measurements were conducted at frequencies ranging from 100 kHz to 10 mHz, with an excitation signal amplitude of 10 Mv. Five tests were conducted for each condition. After electrochemical polarization testing, the corroded specimens were cleaned with ethanol to observe the corrosion conditions of the surface using an optical microscope.

## 3. Results and Discussion

### 3.1. Comparison of Surface Roughness

The roughness of the top surface, 45° surface and side surface of the as-printed samples was measured, respectively. Eight different positions on each sample were measured and their averages were taken to ensure reproducibility of the measurements. Table 4 shows the average $S_{a}$ and $S_{z}$ of the surfaces with each build angle. From Table 4, it can be found that the $S_{a}$ of the top surface is significantly smaller than those of the 45° surface and side surface, while the $S_{a}$ of the 45° surface and side surface had no obvious difference. It can also be found, through measurements, that the $S_{z}$ of the three surfaces is almost the same, without obvious differences.

**Figure 2:**
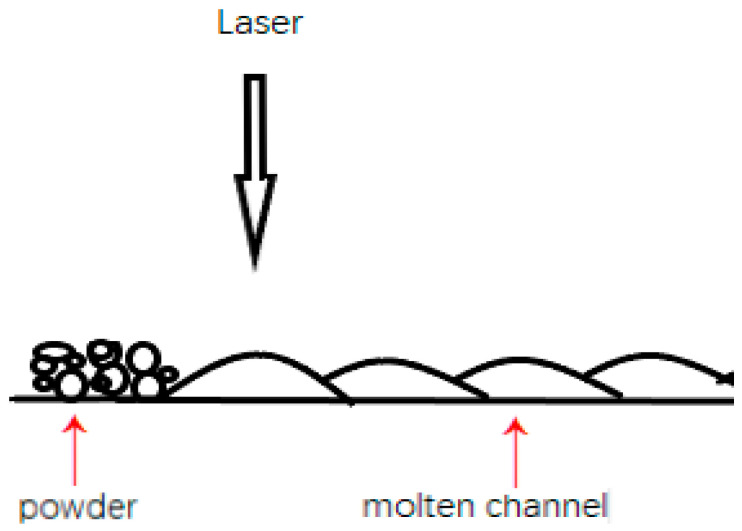
Diagram of molten channel.

**Figure 3:**
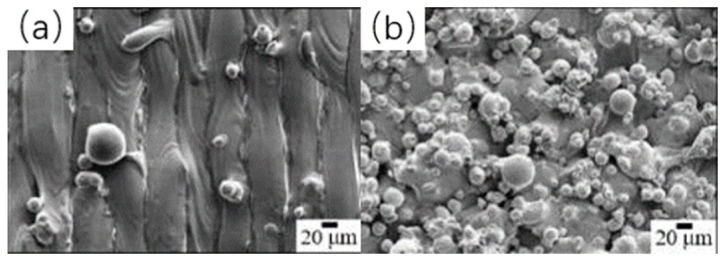
SEM images of the top surface (**a**) and side surface (**b**) of the sample.

**Figure 4:**
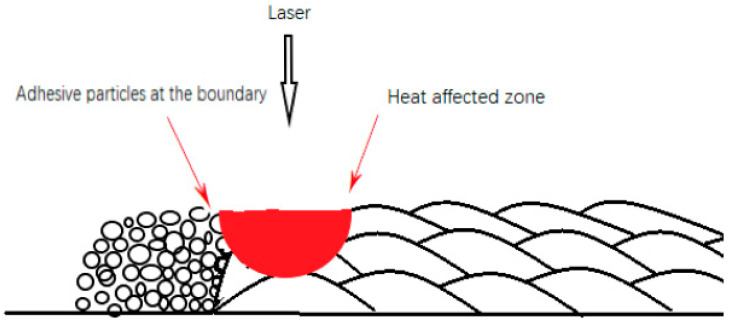
Diagram of incomplete melted powder particles adhered to the surface.

SLM technology is used to make parts by melting powder particles. Due to its own process characteristics, large surface roughness is a significant problem of SLM technology. However, the cause and mechanism of large surface roughness on the top surface and side surface of parts are different. The large surface roughness of the top surface is mainly caused by the molten pool structure formed by laser melting metal powder. SLM is formed from line to plane, that is, the laser is scanned according to the line tracks. The laser scans and melts the metal powder in the form of line tracks, thus forming a series of molten channels. Finally, a complete plane is formed by a series of molten channels. When the powder particles are melted into a liquid state by the laser, under the influence of gravity and surface tension, the molten channels will show an arc with high, middle and low sides, as shown in Figure 2. A series of arc molten channels are connected together to form the uneven topography of the top surface. Figure 3a shows the electron images of the top surface of the sample, from which the channel structure can be clearly observed. The large roughness of the side surface is mainly caused by the adhesion of incomplete fused powder particles to the surface. In the process of SLM forming, the laser is irradiated on the powder bed, and the heat diffuses around it in the form of a Gauss heat source. Therefore, there will be incomplete melted powder particles at the boundary of the heat source. Inside the sample, the laser scans through each track, and the heat source overlaps between adjacent tracks, so that the powder particles inside the sample can be fully melted. However, on the side surface of the sample, the heat source does not overlap, and the powder particles at the boundary of the heat source cannot be completely melted. They will adhere to the surface of the sample by their own melted part, as shown in Figure 4. Figure 3b shows the SEM images of the side surface of the sample, from which it can be observed that a large number of incomplete fused powder particles are adhered to the surface of the sample.

Although there is an obvious molten channel structure on the top surface, the molten channels exist in a smooth arc shape, and the molten channels exist in the unit of lines. While a large number of incomplete fused powder particles are adhered to the side surface in the unit of points. So, the $S_{a}$ of the top surface is significantly smaller than the $S_{a}$ of the side surface. The $S_{a}$ of the 45° surface is similar to the $S_{a}$ of the side surface, indicating that the large roughness of the 45° surface is mainly caused by the adhesion of incomplete fused particles. The reason why the $S_{z}$ of the top surface is not much different from that of the side surface is that there is also a small amount of adhesion of unfused powder particles on the top surface due to sputtering and other factors.

### 3.2. Comparison of Surface Corrosion Resistance

Through electrochemical experiments, the Tafel polarization curves of the top surface, 45° surface and side surface of as-printed samples were determined. Through the analysis of the Tafel polarization curves, the corrosion potential ($E_{corr}$), corrosion current ($I_{corr}$) and pitting potential ($E_{p}$) of each surface were obtained from the curves, which are listed in Table 5.

$E_{corr}$ and $I_{corr}$ reflect the general corrosion of samples, while $E_{p}$ reflects the pitting corrosion of samples. It can be observed from Table 5 that the $E_{corr}$ of the three samples has little difference. While the $I_{corr}$ of the top surface was significantly less than the 45° surface and side surface. Generally, the smaller the value of $I_{corr}$ is, the slower the corrosion rate is, the better the corrosion resistance is. This indicates that the corrosion resistance of the top surface is better than the corrosion resistance of the side surface and 45° surface. From Table 5, it can also be found that the $E_{p}$ of the top surface is significantly higher than the $E_{p}$ of the 45° surface and side surface, while the $E_{p}$ of the 45° surface is slightly lower than the side surface, but with little difference. This variation trend is similar to the variation trend of the roughness of the three surfaces. According to the Galvele pit stability criterion (x.i) [21,22], a larger diffusion length (x) for the anolyte, controlled on the rougher surfaces by the sharp overhung features (10–50 μm deep), would require a lower current density (i) to transition a pit from metastable to stable. Due to the adhesion of a large number of incomplete fused powder particles on the side surface, the surface has a large number of protrusion structures. As a result, the side surface has more potential metastable pitting starting points, and these protrudes are more likely to transition from metastable pitting pits to stable pits. The top surface is mainly composed of a molten channel structure, which is a smooth arc with only a small number of incomplete fused powder particles bonded to the surface. Therefore, there are few protrusion structures on the top surface, so the $E_{p}$ of the top surface is larger and the pitting resistance of the top surface is stronger.

**Figure 5:**
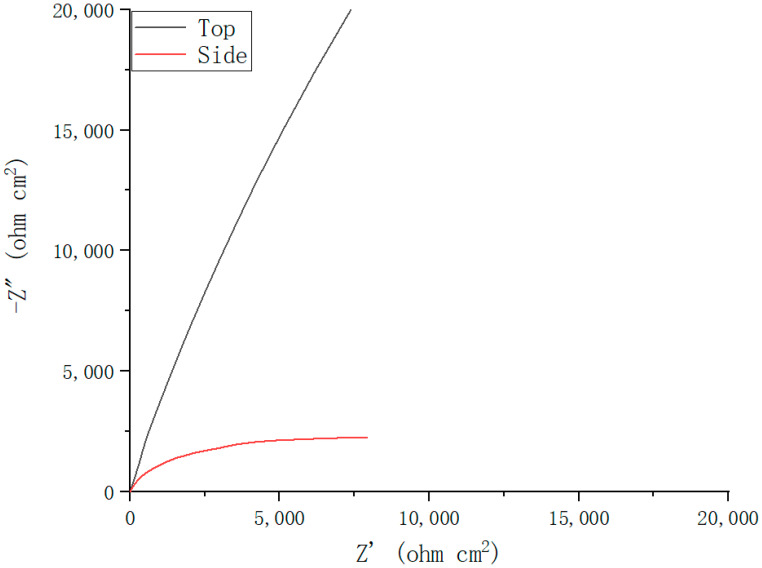
The EIS spectra of top surface and side surface.

**Figure 6:**
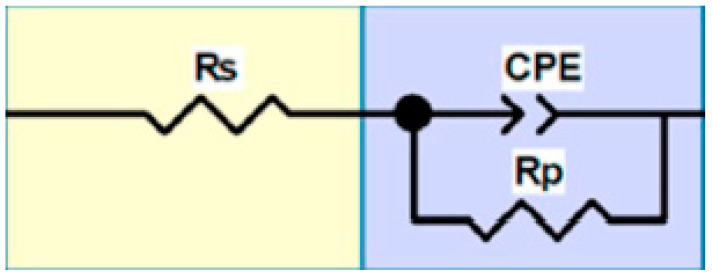
Randle equivalent circuit diagram.

The electrochemical impedance spectroscopy (EIS) spectra are presented in Figure 5. A capacitive arc is shown in the Nyquist plots for the top surface and side surface, which represents the occurrence of corrosion reactions at the stainless steel/electrolyte interface. The radius of a capacitive arc is an important parameter for evaluating the corrosion resistance [23,24], and the larger the radius of the capacitor arc, the better the corrosion resistance. Because the top surface is smoother and the microstructure is more uniform, the corrosion products formed are more evenly adsorbed on the surface. This results in a more stable passivation film and better corrosion resistance. Figure 5 is fitted by the equivalent circuit diagram of Figure 6, and the Rs (the solution resistance), CPE (the constant phase element) and Rp (the polarization resistance) obtained by the fitting are listed in Table 6. A higher Rp value indicates that the passivated film is more stable and, thus, has better corrosion resistance. Therefore, the corrosion resistance of the top surface is better than that of the side surface.

### 3.3. Effect of Polishing on Corrosion Resistance

**Figure 7:**
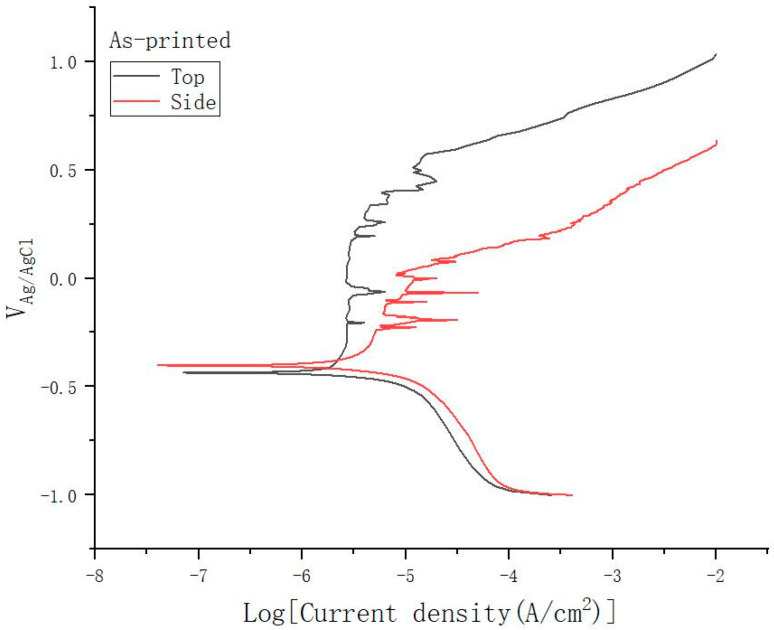
The polarization curve of the as-printed samples.

**Figure 8:**
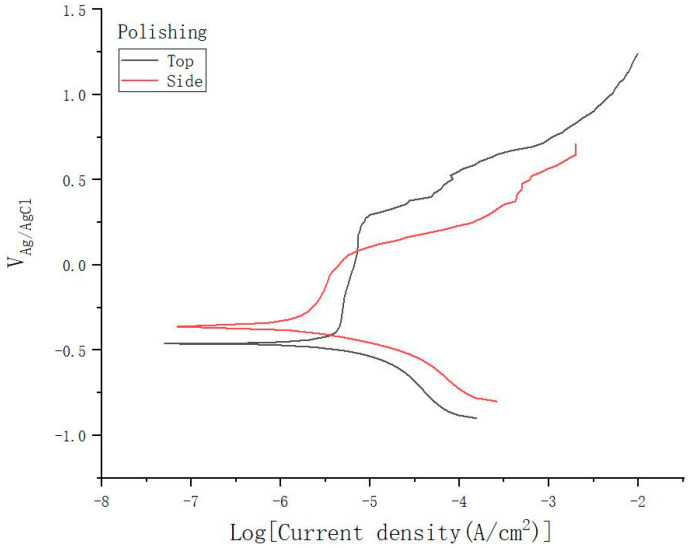
The polarization curve of the polished samples.

It can be found from the polarization curve that the polarization curve of the as-printed surface has many spikes in the passivation area, especially on the side surface, as shown in Figure 7. While this phenomenon is almost not found on the surface of the polished samples, as shown in Figure 8. This is because the as-printed surface has higher metastable pitting activity. Due to the larger surface roughness of the as-printed samples, there are more potential metastable pitting starting points, resulting in more sharp peaks in the passivation area of the polarization curve. At the same time, it is also proved that larger roughness will have more potential metastable pitting starting points, which will increase the possibility of destruction of the passivation film and reduce the corrosion resistance.

Table 7 shows the average and standard deviation of $E_{p}$ values for as-printed and polished samples. The $E_{p}$ value of the polished sample can be obtained from the polarization curve, and the top surface is 0.392 $V_{Ag/AgCl}$ and the side surface is 0.251 $V_{Ag/AgCl}$. 

**Figure 9:**
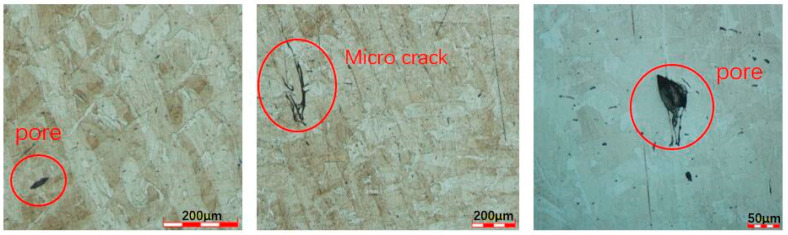
Optical micrograph of pores and microcracks below the surface.

**Figure 10:**
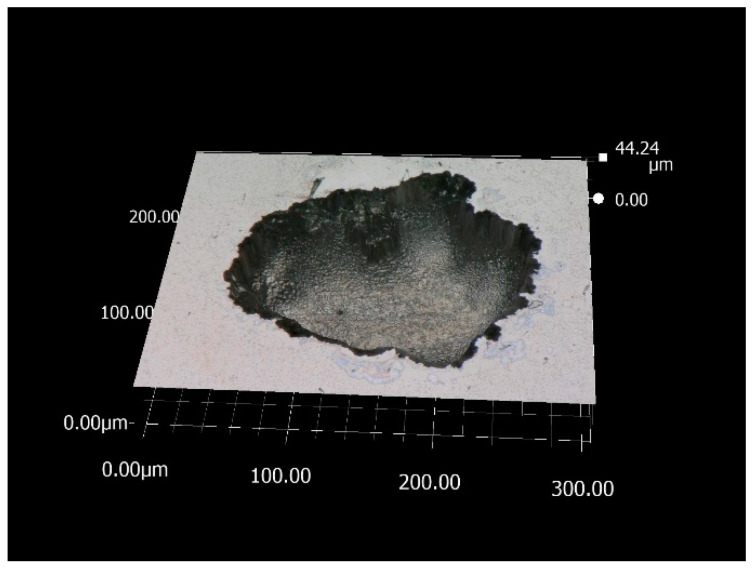
Optical micrograph of pitting pits on the polished samples surface.

It can be found that the $E_{p}$ of the polished side surface is significantly higher than that of the as-printed side surface. Compared with the side surface, the $E_{p}$ of the top surface does not increase significantly after polishing. This is because, after polishing, the surface roughness of the sample is greatly reduced, and the protrusion structure on the side surface is basically eliminated. Therefore, the $E_{p}$ of the side surface of the polished specimen increases, the pitting resistance of the specimen increases, and the passivation film stability increases. However, after polishing, the $E_{p}$ of the top surface does not increase significantly. This is because the polishing exposes defects such as pores and microcracks buried below the surface, as shown in Figure 9. Due to the fault at the bottom of the pore or the accumulation of residual stress, as well as the obstruction of the pore’s own geometric structure to the ion diffusion process, it is easier to gather metal cations in the pore. The hydrolysis of metal cations will lead to the enhancement of the corrosive environment inside the pores. When the environmental corrosivity in the pores increases to a certain extent, it can support the electrochemical activity dissolution of the metal matrix in the inner wall of the pores, so that the pitting corrosion induced by pores will change from metastable growth to stable growth. Therefore, these micro-defects will greatly reduce the pitting resistance of the sample. Moreover, originally, the top surface was a smooth arc melt-channel structure, with only a very small number of unfused particles bonded. Therefore, polishing does little to improve the corrosion resistance of the top surface because of the exposure of defects. In addition, the pitting depth and size of polished samples are generally larger than those of as-built samples. Figure 10 is the micrograph of pitting pits on the polished samples surface, with a depth of 40 μm. 

It can also be found from Table 7 that the standard deviation of the $E_{p}$ value of the as-printed top surface is significantly smaller than that of the as-printed side surface and the polished surface. This is because the as-printed top surface has relatively regular molten pool characteristics and is relatively smooth. Therefore, the standard deviation of the $E_{p}$ value of the as-printed top surface is smaller. The corrosion resistance of the as-printed top surface is more uniform and the dispersion of it is smaller. The as-printed side surface adhered to a large number of incomplete fused powder particles, and the side surface not only has large roughness, but the surface morphology is also complex, without obvious regularity. So, the standard deviation of $E_{p}$ of the as-printed side surface is relatively large. After polishing, the exposed pores and microcracks are dispersed on the surface of the sample, and the sizes of these pores and microcracks are different. These pores and microcracks are important factors to determine the corrosion resistance of the surface. So, after polishing, the corrosion resistance of the sample surface has a large dispersion.

## 4. Conclusions

The reasons for and mechanisms of large surface roughness with different build angles were studied by measuring the roughness of SLM 316L SS. The effects of roughness and polishing on the corrosion resistance of the samples were studied by electrochemical tests. The following conclusions were drawn:

(1) Generally, the surface roughness of SLM parts is larger, and the surface roughness is affected by the build angle. The reason and mechanism that causes the large roughness of the top surface and side surface are different. The large roughness of the top surface is caused by the molten channel structure formed by laser melting metal powder. The large roughness of the side surface is caused by the bonded incomplete fused particles.
(2) Due to the large difference in the roughness between the top surface and the side surface of SLM parts, the corrosion resistance of the top surface and the side surface of SLM parts is quite different. Because the side surface is bonded by a large number of incomplete fused particles, there are a large number of protrusion structures on the side surface. These protrusion structures are the potential starting points of metastable pitting, and it is easier to transition to stable pitting. Therefore, the pitting resistance of the side surface is much lower than that of the top surface.
(3) The surface roughness of SLM parts can be greatly reduced by polishing. For the side surface, the corrosion resistance of the surface can be improved because the protrusion structure on the side surface is basically eliminated. However, for the upper surface, only a small number of incomplete fused particles adhere to the upper surface. Moreover, the polishing exposes defects such as pores and microcracks buried below the surface. These pores and microcracks are more prone to pitting corrosion. Therefore, polishing can improve the corrosion resistance of the side surface. In contrast, the improvement in corrosion resistance of the upper surface is not obvious. At the same time, these pores and microcracks are not evenly distributed on the surface, and their sizes are different, which increases the dispersion of the surface corrosion resistance.

## Figures and Tables

**Table 1:** The particle size distribution of powder.

Cumulative Distribution/%	Particle Size/μm
D10	20.22
D50	32.34
D90	51.84

D10, D50 and D90 refer to the particle size corresponding to the accumulative distribution of 10%, 50% and 90%, respectively.

**Table 2:** Chemical composition of 316L stainless steel powder [6].

Element	Weight%
Fe	Balance
C	0.012
Si	0.69
Mn	1.26
P	0.010
S	0.007
Cr	16.47
Ni	12.72
Mo	2.44
O	0.062

**Table 3:** The processing parameters of SLM.

Laser power (W)	400
Scanning speed (mm/s)	1300
Hatch distance (mm)	0.11
Powder layer thickness (mm)	0.06

**Table 4:** The average of *S_a_* (average surface roughness) and *S_z_* (maximum surface peak/depth) of the three surfaces.

	$S_{a}$ /μm	$S_{z}/\mu m$
Top surface	15.3	181.3
45° suface	18.9	199.5
Side surface	19.1	196.3

**Table 5:** The corrosion potential ($E_{corr}$), corrosion current ($I_{corr}$ ) and pitting potential ($E_{p}$ ) of each surface.

	Top	45°	Side
$E_{corr}$($V_{Ag/AgCl}$)	−0.435	−0.413	−0.402
$I_{corr}$($Acm^{-2}$)	$1.182\times 10^{-5}$	$7.534\times 10^{-5}$	$8.822\times 10^{-5}$
$E_{p}$($V_{Ag/AgCl}$)	0.317	0.183	0.148

**Table 6:** Fitted parameters obtained from the EIS plots.

Specimens	Rs	CPE	Rp
Top	13.77	$3.93\times 10^{-5}$	$137\times 10^{3}$
Side	11.17	$5.61\times 10^{-5}$	$5.16\times 10^{3}$

**Table 7:** The average and standard deviation of $E_{p}$ values for samples.

	The Average of $E_{p}$	The Standard Deviation of $E_{p}$
Top (as-printed samples)	0.317	0.058
Side (as-printed samples)	0.148	0.100
Top (polished samples)	0.372	0.170
Side (polished samples)	0.351	0.147
